# MR Fingerprinting with chemical exchange (MRF-X) to quantify subvoxel T1 and extracellular volume fraction

**DOI:** 10.1186/1532-429X-17-S1-W35

**Published:** 2015-02-03

**Authors:** Jesse I Hamilton, Mark A Griswold, Nicole Seiberlich

**Affiliations:** 1 Biomedical Engineering, Case Western Reserve University, Cleveland, OH, USA; 2 Radiology, Case Western Reserve University, Cleveland, OH, USA

## Background

MR Fingerprinting (MRF) [1] offers a novel approach for quantifying extracellular volume (ECV) in a single scan without contrast agent. Whereas conventional parameter mapping assumes chemical exchange occurs much faster than the experiment time scale, usually on the order of T1, MRF measurements are acquired every TR (less than 12ms). This project investigates the feasibility of a new MRF acquisition termed MRF-X, which takes chemical exchange effects into account, to generate voxel-wise maps of ECV and T1.

## Methods

Signal evolutions were simulated using the Bloch-McConnell equations for two pools with T1_extra_=350ms, T2_extra_=30ms, T1_intra_=1400ms, and T2_intra_=120ms, as suggested in [2]. Four tissues were simulated with ECV values of 25% and 50%, and moderate (k=8.3s^-1^, as in [3]) or fast (k=1000s^-1^) exchange. A standard inversion recovery spin echo experiment was simulated with TE=50ms, TI ranging from 20-3000ms, and complete relaxation between measurements, and then used to fit apparent monoexponential T1 values to mimic native T1 mapping. Additionally, an MRF-X sequence with 500 measurements containing pseudorandom flip angles (0-70 deg) and TRs (9-12ms) was simulated. A sensitivity analysis was also performed by creating a dictionary with the following parameters: T1_intra_ and T1_extra_ 100-2000ms, exchange rate 1-10s^-1^, and ECV 0-100% (T2_extra_=30ms and T2_intra_=120ms were fixed). Noise was added to a randomly selected signal evolution before matching it back to the dictionary. This process was repeated for 1000 entries and SNR levels of 5-100, and the relative error for each parameter was computed as (estimated-actual)/actual. SNR was defined as the maximum in the signal evolution divided by the noise standard deviation.

## Results

**Figure 1:**
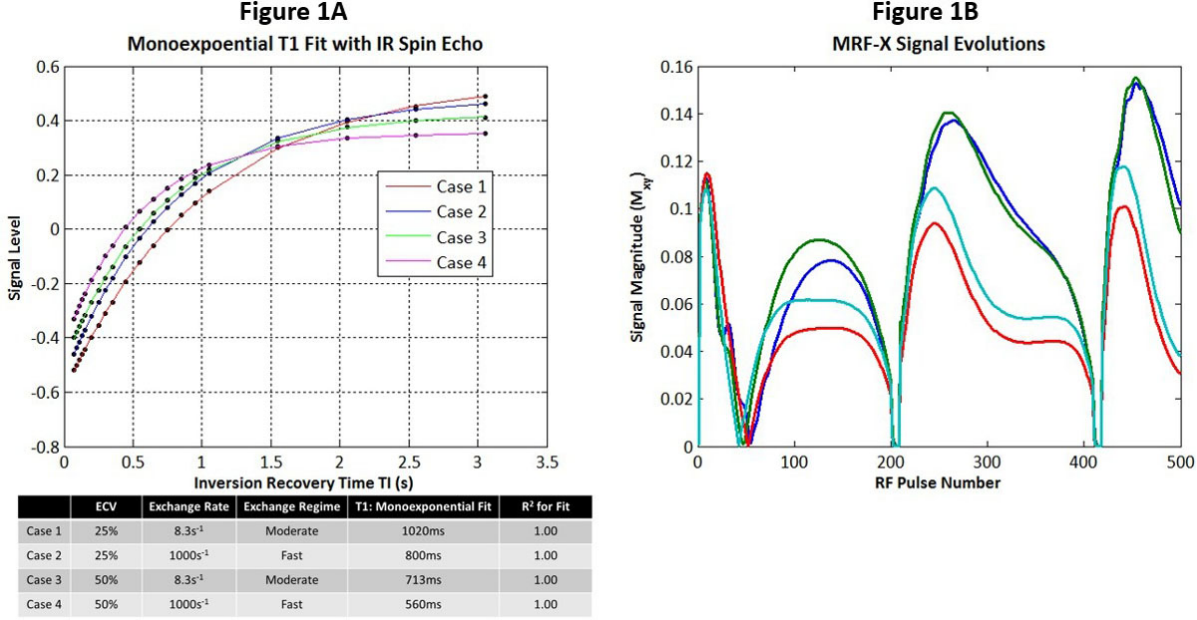
A Monoexponential T1 values fit from a conventional spin echo inversion recovery sequence in four physiologically relevant cases. Figure 1 B: Signal evolutions from a simulated MRF-X sequence are shown for the following tissues: (blue) two compartment species with ECV=25% and moderate exchange, (green) two compartment species with ECV=25% and rapid exchange, (red) single compartment species with T1=1020ms, (cyan) single compartment species with T1=800ms.

**Figure 2:**
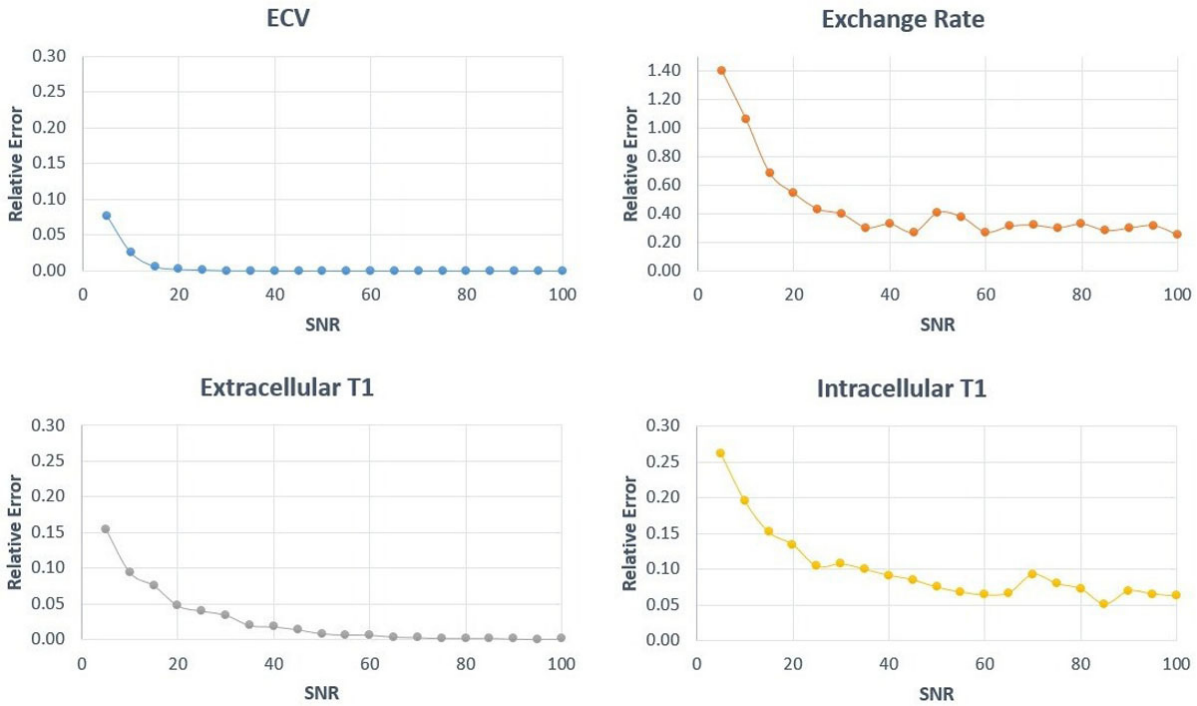
MRF-X sensitivity to ECV, exchange rate, extracellular T1, and intracellular T1 are plotted for different SNR levels.

Figure 1A shows monoexponential fits for T1 using a standard spin echo inversion recovery sequence. Although two compartments are present, a single effective T1 is observed that is different from intracellular or extracellular T1 and varies with exchange rate. Figure 1B shows MRF-X signal evolutions for two-compartment voxels with exchange, as well as single compartment voxels having the effective T1 determined by IR spin echo. The signal evolutions are distinguishable with MRF-X but not with standard T1 mapping. Figure 2 shows that MRF-X displays good sensitivity to ECV, intracellular T1, and extracellular T1, with 10% relative error or less for these parameters above SNR=20. MRF-X is somewhat sensitive to exchange rate with errors below 40% at SNR=30 and 25% at SNR=100; sequence optimization could potentially improve the accuracy in fitting this parameter.

## Conclusions

MRF-X has the potential to quantify subvoxel relaxation parameters and volume fractions when chemical exchange is present and could be used to map ECV in a single scan without exogenous contrast agent.

## Funding

NIH/NIBIB R00EB011527 and Siemens Medical Solutions.
